# Supplementary material for: What influences parental decisions about antibiotic use with their children: A qualitative study in rural Australia
Source: PLoS One. 2023 Jul 19;18(7):e0288480. doi: 10.1371/journal.pone.0288480 (PMC10355396; doi:10.1371/journal.pone.0288480)
Supplement: S1 Checklist — (PDF) [file pone.0288480.s001.pdf]

COREQ Checklist

No	Item	Guide questions/description	Page / evidence
Domain 1: Research team and reflexivity			
Personal Characteristics			
1.	Interviewer/ facilitator	Which author/s conducted the interview or focus group?	Methods – Data collection (pg. 7)
2.	Credentials	What were the researcher's credentials? *E.g. PhD, MD*	Author affiliations (pg. 1)<br>Methods – Data collection (pg. 7)
3.	Occupation	What was their occupation at the time of the study?	Methods – Data collection (pg. 7)
4.	Gender	Was the researcher male or female?	Methods – Data collection (pg. 7)
5.	Experience and training	What experience or training did the researcher have?	Methods – Data collection (pg. 7)
Relationship with participants			
6.	Relationship established	Was a relationship established prior to study commencement?	No
7.	Participant knowledge of the interviewer	What did the participants know about the researcher? e.*g. personal goals, reasons for doing the research*	Methods – Ethics<br>*Participant Information Sheet*<br>(pg. 8)
8.	Interviewer characteristics	What characteristics were reported about the interviewer/facilitator? e.g. *Bias, assumptions, reasons and interests in the research topic*	Methods – Ethics<br>*Participant*

No	Item	Guide questions/description	Page / evidence
			Information Sheet - which provided details of the interests and characteristics of the researcher(s).<br>(pg. 8)
Domain 2: study design			
Theoretical framework			
9.	Methodological orientation and Theory	What methodological orientation was stated to underpin the study? *e.g. grounded theory, discourse analysis, ethnography, phenomenology, content analysis*	Introduction (pg. 4)<br>Methods – Analysis (pg. 8)
Participant selection			
10.	Sampling	How were participants selected? *e.g. purposive, convenience, consecutive, snowball*	Methods – Study population and recruitment (pg. 6)
11.	Method of approach	How were participants approached? e.*g. face-to-face, telephone, mail, email*	Methods – Study population and recruitment (pg. 6)
12.	Sample size	How many participants were in the study?	Results (pg. 8)
13.	Non-participation	How many people refused to participate or dropped out? Reasons?	Methods – Study population and recruitment (pg. 6) and Data collection (pg. 7).
Setting			

No	Item	Guide questions/description	Page / evidence
14.	Setting of data collection	Where was the data collected? e.*g. home, clinic, workplace*	Methods – Study design (pg. 5)
15.	Presence of non-participants	Was anyone else present besides the participants and researchers?	Methods – Data collection (pg. 7)
16.	Description of sample	What are the important characteristics of the sample? *e.g. demographic data, date*	Study population and recruitment (pg. 6)<br>Results – Table 1. Participant characteristics (pg. 8)
Data collection			
17.	Interview guide	Were questions, prompts, guides provided by the authors? Was it pilot tested?	Methods – Data collection (pg. 7)<br>Supporting Information – S1 Focus Group Guide
18.	Repeat interviews	Were repeat interviews carried out? If yes, how many?	Not applicable
19.	Audio/visual recording	Did the research use audio or visual recording to collect the data?	Methods – Data collection (pg. 7)
20.	Field notes	Were field notes made during and/or after the interview or focus group?	Methods – Data collection (pg. 7)
21.	Duration	What was the duration of the interviews or focus group?	Methods – Data collection (pg. 7)
22.	Data saturation	Was data saturation discussed?	Methods – Data collection (pg. 7)

No	Item	Guide questions/description	Page / evidence
23.	Transcripts returned	Were transcripts returned to participants for comment and/or correction?	No.<br>Clear audio/visual recordings were obtained and stored securely in accordance with ethics approval.
Domain 3: analysis and findings			
Data analysis			
24.	Number of data coders	How many data coders coded the data?	Supporting Information – S2 Data Analysis Protocol
25.	Description of the coding tree	Did authors provide a description of the coding tree?	Supporting Information – S2 Data Analysis Protocol
26.	Derivation of themes	Were themes identified in advance or derived from the data?	Methods – Analysis (pg. 8)<br>Supporting Information – S2 Data Analysis Protocol
27.	Software	What software, if applicable, was used to manage the data?	Supporting Information – S2 Data Analysis Protocol
28.	Participant checking	Did participants provide feedback on the findings?	No.<br>Researcher triangulation was

No	Item	Guide questions/description	Page / evidence
			used during phases of data analysis.<br>Supporting Information – S2 Data Analysis Protocol
Reporting			
29.	Quotations presented	Were participant quotations presented to illustrate the themes / findings? Was each quotation identified? e.*g. participant number*	Yes - Results (pg. 10-21)
30.	Data and findings consistent	Was there consistency between the data presented and the findings?	Results (pg. 9-21)
31.	Clarity of major themes	Were major themes clearly presented in the findings?	Results – (pg. 9-21)<br>Table 2. Themes and sub-themes (pg. 9)
32.	Clarity of minor themes	Is there a description of diverse cases or discussion of minor themes?	Results – (pg. 9-21)<br>Table 2. Themes and sub-themes (pg. 9)
