# Supplementary material for: What influences parental decisions about antibiotic use with their children: A qualitative study in rural Australia
Source: PLoS One. 2023 Jul 19;18(7):e0288480. doi: 10.1371/journal.pone.0288480 (PMC10355396; doi:10.1371/journal.pone.0288480)
Supplement: S1 File — (PDF) [file pone.0288480.s002.pdf]

S1. Focus Group Guide

1) *What do you think are some of the benefits of giving your child antibiotics?*

- **Prompt:** *When do you think antibiotics should be used and when should they not be used?*

2) *What concerns do you have about giving your child antibiotics?*

- **Prompt:** *Have you heard about antibiotic resistance or superbugs? What do you think about it?*

3) *What advice do you consider when making decisions about giving your child antibiotics?*

- **Prompt:** *Do you talk to other people in your social group or family about giving your child antibiotics? If so, what do they suggest?*

- **Further prompting (if appropriate):** *When is it more likely that you would consult with your family or social group about using antibiotics for your child?*

4) *Have you ever gone into a consultation with a doctor about your child with the intention of getting a prescription for antibiotics?*

- **Further prompting (if appropriate):** *If yes, why did you think that an antibiotic was necessary?*

5) Sometimes people don't follow the doctor's instructions precisely when giving their children antibiotics. Have you ever varied from the precise instructions?

- **If yes, prompt:** *What influenced your decision to not follow the instructions precisely?*

6) What are your thoughts about keeping leftover antibiotics for later use?

- **Prompt:** *When do you use leftover antibiotics?*

7) What are your thoughts about sharing your child's antibiotics with others?

- **Prompt:** *What influences your decision to share leftover antibiotics?*

8) How accessible are health services when your child is ill?

Follow-up: *Does this influence your decisions about giving your child leftover antibiotics or antibiotics given to you by a friend or family member?*

- **Prompt:** *In what way does this influence you?*
