# Supplementary material for: What influences parental decisions about antibiotic use with their children: A qualitative study in rural Australia
Source: PLoS One. 2023 Jul 19;18(7):e0288480. doi: 10.1371/journal.pone.0288480 (PMC10355396; doi:10.1371/journal.pone.0288480)
Supplement: S2 File — (PDF) [file pone.0288480.s003.pdf]

Data Analysis Protocol

Phases of Thematic Analysis	Data Analysis Steps
**Phase 1: Familiarising yourself with your data**	Transcripts were read and re-read by SM for data immersion and familiarisation to identify similar response patterns. This included reflective journaling and documenting thoughts about data patterns, codes, possible themes and reviewing observational notes.
**Phase 2: Generating initial codes**	Initial coding categories were constructed by SM, which captured the content of mother's responses to organise the data into meaninful groups to assist with collation. Coding was conducted manually, using a tally system and highlighting the data. Qualitative analysis software was not used to organise the data. Analytic thoughts were documented and supported with illustrative extracts.
**Phase 3: Searching for Themes**	Codes were entered into a Microsoft Excel document by SM with extracted quotations under each code. Codes were compiled using an abductive approach performed by SM independently. This involved an iterative process of reviewing and reflecting on the data and observed similarities between codes, to generate themes and sub-themes that were both data-driven, and considered evidence of theoretical variables. Visual representations of the initial theme hierarchy were developed to assist with connecting preliminary themes and sub-themes.
**Phase 4: Reviewing Themes**	Researcher triangulation was a method used amongst the research team involving the participation of 3 researchers (SM, SP and MB) at this stage of data analysis. During meetings, codes with data extracts, constituent and superordinate themes were presented and discussed amongst team members to provide multiple observations, interpretations, suggestions and conclusions for the data. Any disagreement in the interpretation of the data was resolved through team discussion and sharing ideas until agreement was reached.
**Phase 5: Defining and Naming Themes**	Further collaboration amongst the researchers (SM, MB, SP) occurred to refine themes by relabelling and finalising the theme hierarchy until consensus was obtained. Regular team discussion occurred to reach concurrence regarding the names of the final themes and sub-themes to be presented for analysis. Any changes to the theme hierarchy were documented.
**Phase 6: Producing the Report**	Using descriptive quotations from the transcripts an analytical narrative of the themes generated from the data analysis was developed, and is presented in the results section.
